# Bis(1-pyrenylmethyl)-2-benzyl-2-methyl-malonate as a Cu^2+^ Ion-Selective Fluoroionophore

**DOI:** 10.3390/molecules22091415

**Published:** 2017-08-25

**Authors:** Takayo Moriuchi-Kawakami, Youji Hisada, Akihisa Higashikado, Tsubasa Inoue, Keiichi Fujimori, Toshiyuki Moriuchi

**Affiliations:** 1 Department of Applied Chemistry, Faculty of Engineering, Osaka Institute of Technology, 5-16-1 Omiya, Asahi, Osaka 535-8585, Japan; oitrecogchem@yahoo.co.jp (Y.H.); m1m17518@st.oit.ac.jp (A.H.); m1m17504@st.oit.ac.jp (T.I.); keiichi.fujimori@oit.ac.jp (K.F.); 2 Department of Applied Chemistry, Graduate School of Engineering, Osaka University, 2-1 Yamada-oka, Suita, Osaka 565-0871, Japan; moriuchi@chem.eng.osaka-u.ac.jp

**Keywords:** bispyrenyl malonate, Cu^2+^ ion-selective, fluoroionophore, CH···π Interaction, π···π interaction, X-ray crystallographic analysis

## Abstract

A new malonate possessing two pyrene moieties was synthesized as a fluoroionophore, and its structure and fluorescence spectroscopic properties were investigated. When excited at 344 nm in acetonitrile/chloroform (9:1, *v*/*v*), the synthesized bispyrenyl malonate has the fluorescence of intramolecular excimer (λ_em_ = 467 nm) emissions and not a pyrene monomer emission (λ_em_ = 394 nm). A large absolute fluorescence quantum yield was obtained in the solid state (Φ_PL_ = 0.65) rather than in solution (Φ_PL_ = 0.13). X-ray crystallography analysis clarified the molecular structure and alignment of the bispyrenyl malonate in the crystal phase, elucidating its fluorescence spectroscopic properties. Such analysis also suggests there are intramolecular C–H···π interactions and intermolecular π···π interactions between the pyrenyl rings. Interestingly, the synthesized bispyrenyl malonate exhibits excellent fluorescence sensing for the Cu^2+^ ion. Remarkable fluorescence intensity enhancement was only observed with the addition of the Cu^2+^ ion.

## 1. Introduction

In recent years, research on ion sensing by fluoroionophores has attracted considerable attention [1,2,3,4,5]. In fact, there have been many reports on ion sensing for the Cu^2+^ ion since it is not only a toxic environmental pollutant but also an essential trace element in biological systems [6,7,8,9]. The Cu^2+^ ion is a well-known paramagnetic ion with an unfilled *d* orbital and can strongly quench the fluorescence of a fluorophore in its proximity via electron or energy transfer [10]. Therefore, the quenching of the fluorescence emission derived from the Cu^2+^ ion has mostly been reported in literature on ion sensing for the Cu^2+^ ion by fluoroionophores [4,11,12,13,14,15,16]. Nevertheless, a few reports on fluorescent enhancement with the Cu^2+^ ion are available [17,18,19,20,21].

Most fluoroionophores for cation sensing can be constructed with the recognition site having a fluorescent moiety [9]. Pyrenes are used extensively as a fluorescent moiety due to their emission properties [22,23,24,25,26]. Interestingly, fluorescent molecules with more than one pyrene moiety not only have pyrene monomer emissions but also pyrene excimer emissions due to the strong π···π interactions between the two pyrene moieties [27,28]. Generally, the pyrene excimer emission is observed in the longer wavelength region and is stronger than the pyrene monomer emission. If an efficient excimer emission signal is utilized for ion sensing, it would provide a sensitive detection method for the Cu^2+^ ion. In fact, ion sensing accompanied by the pyrene excimer emission signal has been reported for various ions such as H^+^, Ca^2+^, Zn^2+^ , Zr^4+^, In^3+^, Pb^2+^, and I^−^ ions [29,30,31,32,33,34,35,36,37]. There are some reports in which pyrene derivatives detect the Cu^2+^ ion by a ratiometric fluorescent response with both monomer and excimer emissions [38,39,40,41,42], and where pyrene derivatives selectively recognize Cu^2+^ via the excimer emission enhancement [43,44,45,46,47]. However, for more highly sensitive sensing, improvement of the fluorescence quantum yield is necessary. In our previous studies, it was demonstrated that sandwich-type ion recognition compounds indicate excellent ion selectivity and that malonate is an excellent spacer for such compounds [48,49,50]. The substituents introduced into the C2-position of the malonate spacers affect the dihedral angles between the two ion recognition moieties, although the introduced substituents are spatially distant from the moieties [48]. In this study, we have designed and synthesized a new malonate possessing two pyrene moieties as a fluoroionophore. The dihedral angles between the two pyrenyl rings of the fluorescent probe are controlled by the substituents introduced into the C2-position of the malonate spacers, thus leading to the improvement of the pyrene excimer emissions and fluorescence quantum yield. Here, we have reported the synthesis, fluorescence spectroscopic properties, and the structure of the bis (1-pyrenemethyl)-2-benzyl-2-methylmalonate **1**.

## 2. Results and Discussion

### 2.1. Synthesis

**Scheme 1:**
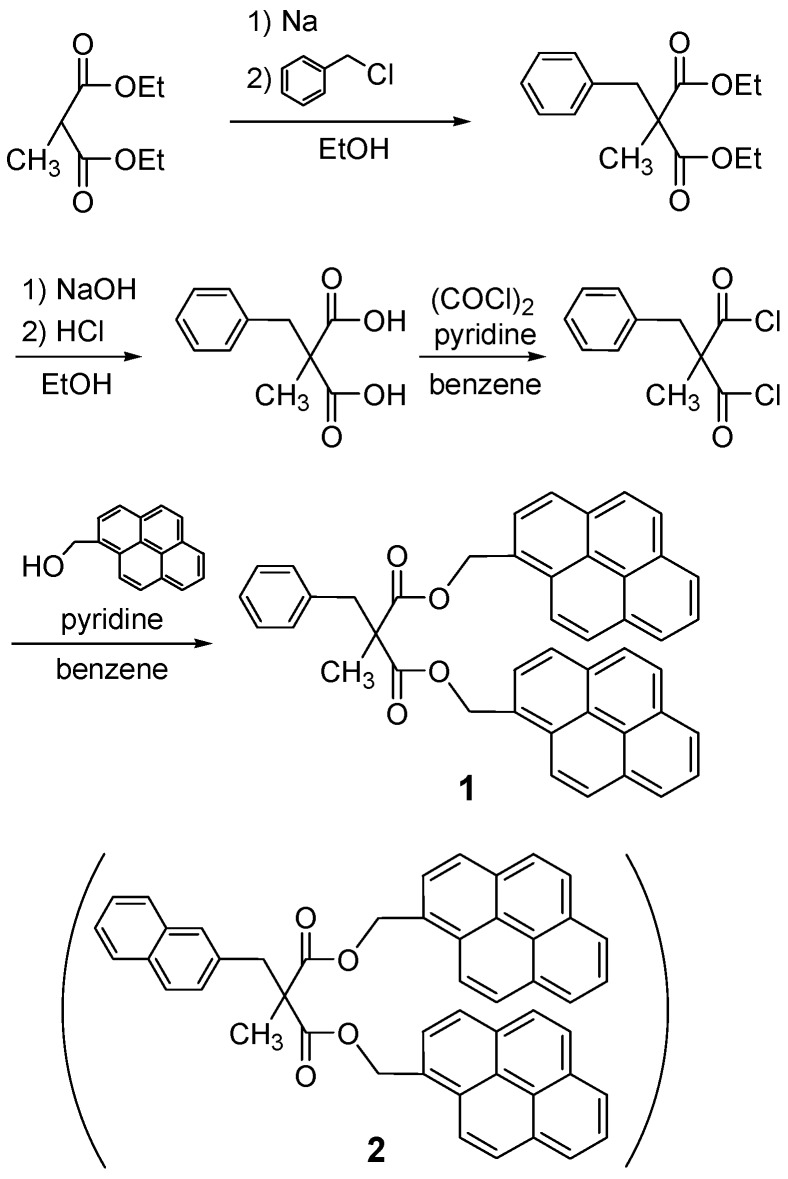
Synthesized bispyrenyl malonates **1** and **2**.

The synthesis of bispyrenyl malonate **1** proceeded from the starting 2-benzyl-2-methyl-malonic acid diethyl ester obtained by the introduction of the benzyl group to commercially available methylmalonic acid diethyl ester. The synthetic route of bispyrenyl malonate **1** is depicted in Scheme 1. Disubstituted malonic acid dichloride was synthesized by the reaction of the corresponding disubstituted malonic acid with (COCl)_2_ in benzene [49]. Subsequently, the reaction of the disubstituted malonic acid dichloride with 1-pyrenylmetanol in benzene led to the desired bispyrenyl malonate **1** in a 66% isolated yield. The thus-obtained bispyrenyl malonate **1** (C_45_H_32_O_4_) was fully characterized by ^1^H-NMR, ^13^C-NMR, FTIR, and HRMS. Detailed data on bispyrenyl malonate **1** are described in the Experimental Section. For comparison, bispyrenyl malonate **2** was prepared from the corresponding 2-methyl-2-naphthalenylmethyl-malonic acid diethyl ester.

### 2.2. Absorption and Fluorescence Properties

**Figure 1:**
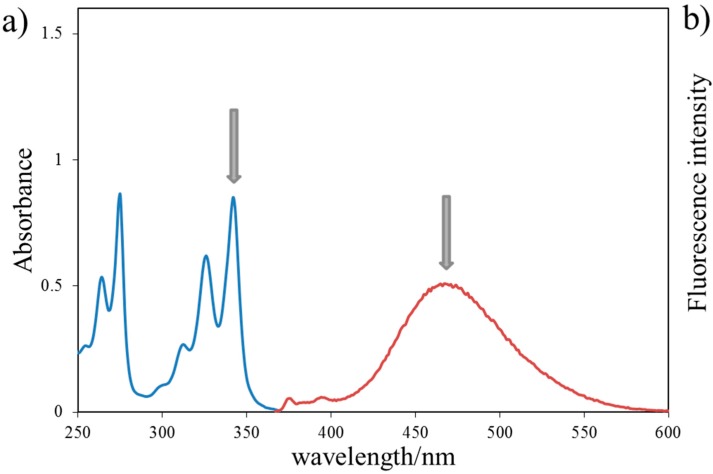
(**a**) UV absorption (blue line) and (**b**) Fluorescence spectra (red line) of bispyrenyl malonate **1** (10 μM) in acetonitrile/chloroform (9:1, *v*/*v*) (excitation wavelength: λ_ex_ = 344 nm).

The UV-absorption spectra and fluorescence emission spectra of bispyrenyl malonate **1** (1.0 × 10^–5^ M) measured using an acetonitrile/chloroform (9:1, *v*/*v*) solution are shown in Figure 1. The maximum absorption bands of bispyrenyl malonate **1** are located at 342 and 326 nm (Figure 1a). Bispyrenyl malonate **1** itself shows a broad fluorescence band at 467 nm (excitation wavelength: λ_ex_ = 344 nm) (Figure 1b). It indicates that bispyrenyl malonate **1** has the fluorescence of intramolecular excimer (λ_em_ = 467 nm) emissions and not a pyrene monomer emission (λ_em_ = 394 nm) even when bispyrenyl malonate **1** is present in the solution. The absolute quantum yields of bispyrenyl malonate **1** at room temperature were also recorded on an absolute PL quantum yield (Φ_PL_) measurement system. The absolute fluorescence quantum yields of bispyrenyl malonate **1** were Φ_PL_ = 0.13 in acetonitrile/chloroform (9:1, *v*/*v*) solution and Φ_PL_ = 0.65 in solid (excitation wavelength: λ_ex_ = 344 nm). Interestingly, the large absolute fluorescence quantum yield of bispyrenyl malonate **1** could be obtained in the solid phase rather than in solution.

### 2.3. X-ray Structural Studies

**Figure 2:**
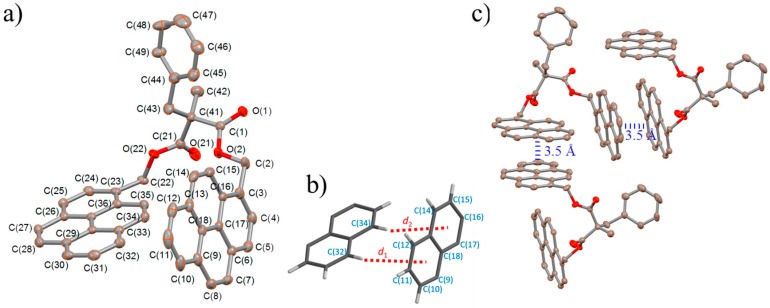
(**a**) Molecular structure; (**b**) schematic representation of the intramolecular C–H···π interactions and (**c**) alignment of bispyrenyl malonate **1** determined by X-ray crystallographic analysis (40% probability of thermal ellipsoid plots).

**Figure 3:**
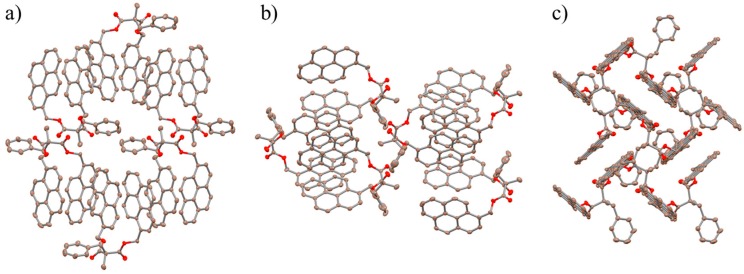
(**a**) Projection down the *a* axis; (**b**) projection down the *b* axis; and (**c**) projection down the *c* axis of the molecular packing of bispyrenyl malonate **1**.

X-ray crystallography analysis clarified the molecular structure and alignment of bispyrenyl malonate **1** in the crystal phase, as shown in Figure 2 and Figure 3. The structure was determined in the orthorhombic space group *P*2_1_2_1_2_1_ (no. 19) and anisotropic displacement parameters were applied for the ordered non-H atoms in the structures. The X-ray crystallographic data are summarized in Table 1 and the selected bond lengths and angles are listed in Table 2.

Structural characterizations of bispyrenyl malonate **1** were carried out by single-crystal X-ray structure determination to elucidate the fluorescence spectroscopic properties. As shown in Figure 2b, the calculated positions of the hydrogen atoms on the pyrenyl C (32) and C (34) atoms are practically facing the π-electrons of the other pyrenyl ring. The interaction distances (*d*_1_ and *d*_2_) between the hydrogens and the pyrenyl moiety are 2.58 and 2.60 Å, suggesting intramolecular C–H···π interactions in the crystal structure (edge-to-face interactions). These distances associated with the C–H···π interactions are sufficiently close to form an excimer [51]. The dihedral angles between the two pyrenyl moieties resulting from the C–H···π interactions are 83.05(6)°. The results of this single crystal X-ray study on bispyrenyl malonate **1** support the present fluorescence spectroscopic properties. Bispyrenyl malonate **1** in solution exhibits the fluorescence of intramolecular pyrene excimer emission (λ_em_ = 467 nm) due to the structural influence from the intramolecular C–H···π interaction between the two pyrene moieties.

Furthermore, pyrenyl moieties also participate in intermolecular π···π interactions with each of their neighboring molecules. Figure 2c shows that the pyrenyl moiety is present in a face-to-face manner with an interplanar distance of ca. 3.5 Å between the two pyrenyl moieties. The distance is within the range of the typical distance for π···π interactions (3.5 Å) [52]. The molecular packing diagrams of bispyrenyl malonate **1** in Figure 3 indicate that the molecules are linked by the π·· π stacking formed between the pyrenyl moieties with adjacent molecules. As shown in Figure 3c, pyrenyl moieties arrange in a herringbone motif that combines edge-to-face contacts (interaction distance = ca. 2.6 Å), the face-to-face π···π stacking (interaction distance = ca. 3.5 Å), and the offset π ·· π stacking (center-center distance = 9.51 Å) [53]. The intramolecular C–H···π interaction and the intermolecular π·· π interaction stabilize the structure of bispyrenyl malonate **1**. A large absolute fluorescence quantum yield of bispyrenyl malonate **1** was obtained in the solid state (Φ_PL_ = 0.65) rather than in solution (Φ_PL_ = 0.13) due to the influence of the intramolecular C–H···π interaction and the intermolecular π·· π interaction in the solid state.

### 2.4. Fluorescence Response

**Figure 4:**
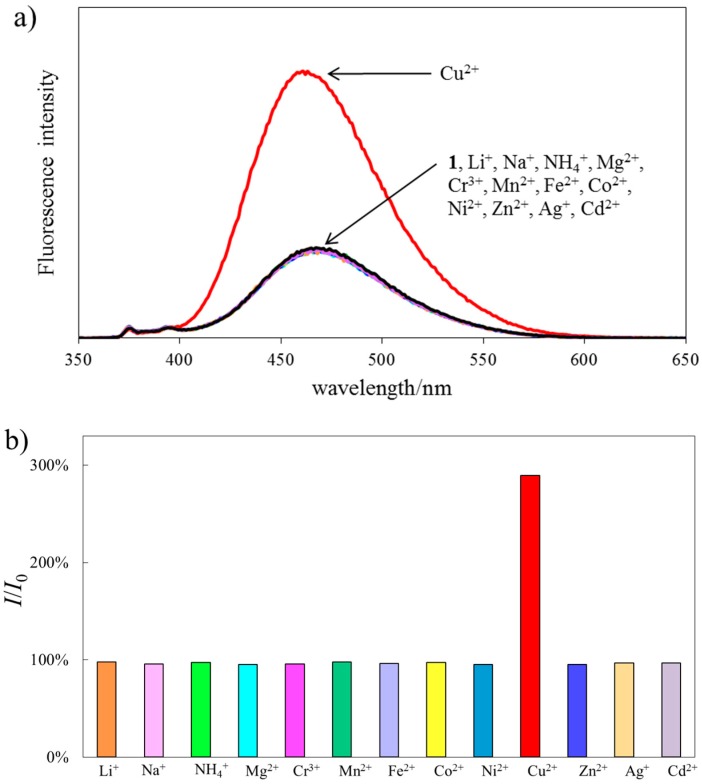
(**a**) Fluorescence spectra and (**b**) ratio of the fluorescence intensities monitored at 467 nm of bispyrenyl malonate **1** (10 μM) in the presence of 1 equiv. of Li^+^, Na^+^, NH_4_^+^, Mg^2+^, Cr^3+^, Mn^2+^, Fe^2+^, Co^2+^, Ni^2+^, Cu^2+^, Zn^2+^, Ag^+^, and Cd^2+^ in acetonitrile/chloroform (9:1, *v*/*v*) (excitation wavelength: λ_ex_ = 344 nm).

The fluorescence response to various cations (Li^+^, Na^+^, NH_4_^+^, Mg^2+^, Cr^3+^, Mn^2+^, Fe^2+^, Co^2+^, Ni^2+^, Cu^2+^, Zn^2+^, Ag^+^, and Cd^2+^) of bispyrenyl malonate **1** was examined. The fluorescence spectra were recorded in acetonitrile/chloroform (9:1, *v*/*v*) solutions at a concentration of 1.0 × 10^–5^ M (excitation wavelength: λ_ex_ = 344 nm). Figure 4a shows the fluorescence spectra of bispyrenyl malonate **1** in the absence or presence of 1 equiv. of each respective cation. The maximal emission peaks of bispyrenyl malonate **1** are located at 467 nm. Only the addition of the Cu^2+^ ion to the solution of bispyrenyl malonate **1** led to an enhancement of the fluorescence intensity. The maximal emission peak of bispyrenyl malonate **1** at 467 nm slightly shifted (to 463 nm) in the presence of 1 equiv. of the Cu^2+^ ion. As shown in Figure 4b, the fluorescence intensity at 467 nm in the presence of 1 equiv. of the Cu^2+^ ion was stronger than that of bispyrenyl malonate **1** itself. In addition, no fluorescence intensities of the solutions of bispyrenyl malonate **1** and 1 equiv. of the Cu^2+^ ion were changed in the presence of 1 equiv. of other metal ions (Figure S1). These results demonstrate that bispyrenyl malonate **1** exhibits high Cu^2+^ ion-selectivity even if in the presence of competitive cations. Although the addition of Cu^2+^ ions (from 0 to 100 equiv.) to the solution of bispyrenyl malonate **1** led to an increase in the fluorescence intensities of bispyrenyl malonate **1**, no quantitative relationship between the fluorescence intensities of bispyrenyl malonate **1** and the concentrations of the Cu^2+^ ions was observed.

**Figure 5:**
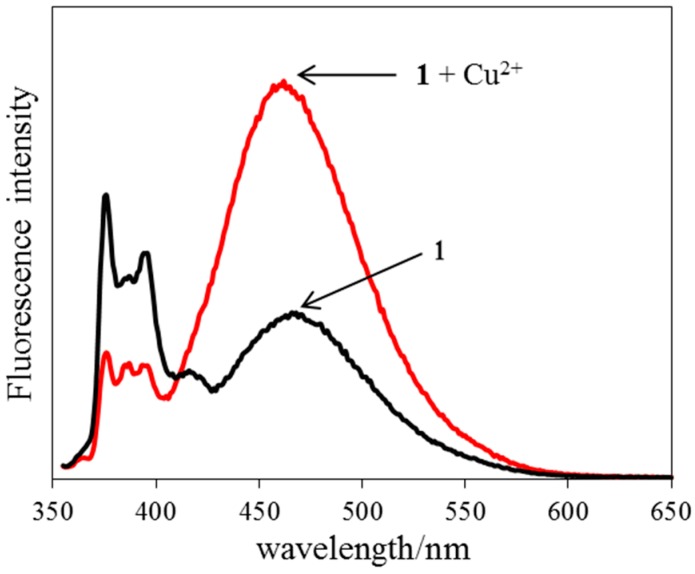
Fluorescence spectra of bispyrenyl malonate **1** (10 μM) in the presence of 1 equiv. of Cu^2+^ in acetonitrile/chloroform/methanol/water (7:1:1:1, *v*/*v*) (excitation wavelength: λ_ex_ = 344 nm).

Based on the solubility of bispyrenyl malonate **1**, a mixed solution (acetonitrile/chloroform/methanol/water = 7:1:1:1, *v*/*v*) was applied as the aqueous solution for further investigations. Under this aqueous condition, the proposed bispyrenyl malonate **1** showed a large fluorescence of the pyrene monomer emission and a small fluorescence of the pyrene excimer emission. The addition of the Cu^2+^ ions decreased the fluorescence of the pyrene monomer emission and increased the fluorescence of the pyrene excimer emission (Figure 5). It is notable that specific fluorescence responses of bispyrenyl malonate **1** to the Cu^2+^ ions were exhibited even if in aqueous conditions.

In our previous studies, it was demonstrated that the malonate spacer possessing the methyl and naphthalenylmethyl groups as C2-position introduced substituents also increased the ion-selectivity of the sandwich-type ion recognition compounds [49]. Thus, bispyrenyl malonate **2** possessing the methyl and naphthalenylmethyl groups as substituents was also prepared by a similar method (Scheme 1) and, for comparison, the fluorescence spectra of bispyrenyl malonate **2** were measured in the absence and presence of 1 equiv. of each respective cation (Figure S2). Fluorescence spectra findings on bispyrenyl malonates **1** and **2** demonstrated that they exhibit similar Cu^2+^ ion-selectivity. However, the ion-selectivity against the examined cations of bispyrenyl malonate **1** was slightly superior to that of bispyrenyl malonate **2**. This is assumed to be because the substituents introduced into the C2-position of the malonate spacers affect the dihedral angles between the two pyrenyl rings, although the introduced substituents are spatially distant from the pyrenyl rings.

Fluorescence intensity enhancement of the bispyrenyl malonates by the Cu^2+^ ions could be interpreted as follows: the binding of the Cu^2+^ ion to ester moieties of a bispyrenyl malonate is considered to shorten the distance between the intramolecular pyrenyl rings, resulting in fluorescence intensity enhancement of the pyrene excimer emissions. Such fluorescence intensity enhancement was also influenced by the counter anions, i.e., fluorescence intensity enhancement of bispyrenyl malonate **2** by the addition of the Cu^2+^ ions in the case of nitrate was markedly weaker than that in the case of perchlorate.

## 3. Experimental Section

### 3.1. Reagents and Chemicals

All reagents were commercially available in the highest grade and used for the syntheses of malonates as such unless otherwise specified. Ethyl alcohol and pyridine were dried over molecular sieve 4 Å. Benzene was dried over sodium and distilled. All reactions were carried out under dry nitrogen. Acetonitrile was supplied from Wako Pure Chemical Industries Ltd. (Chuo-ku, Osaka, Japan) in the spectrochemical analysis grade for the absorption and fluorescence spectrometries. Chloroform was supplied from Wako Pure Chemical Industries, Ltd. in high performance liquid chromatography grade. Metal cations were added to a solution of a malonate derivative as perchlorate salts for the absorption and fluorescence spectrometries.

### 3.2. Apparatus

The ^1^H- and ^13^C-NMR spectra were recorded at 300 or 400 and 75 or 100 MHz, respectively. Samples for NMR spectra were examined in CDCl_3_ solutions at 25.0 °C on a Varian 300 MHz (XL-300) (Agilent Technologies Inc., Santa Clara, CA, USA) or a JEOL 400 MHz (JMTC-400) (JEOL Ltd., Akishima, Tokyo, Japan) NMR spectrometers. Chemical shifts are given in δ (ppm) relative to deuterated solvents (^13^C-NMR) or to TMS (^1^H-NMR) as an internal standard. IR spectra were run in KBr discs on a Shimazu FTIR-8600 spectrometer (Shimazu Corporation, Nagagyo-ku, Kyoto, Japan). High-resolution mass (HRMS) spectra (positive mode of EI mass) were recorded on a JEOL JMS-DX-303 (JEOL Ltd., Akishima, Tokyo, Japan). UV-Vis spectra were recorded on a Shimazu MPS-2000 Spectrophotometer (Shimazu Corporation, Nagagyo-ku, Kyoto, Japan). Fluorescence emission spectra were recorded on a Shimazu RF-5300PC(S) Luminescence Spectrometer (Shimazu Corporation, Nagagyo-ku, Kyoto, Japan). Absolute fluorescence quantum yields were determined with a Hamamatsu Photonics Quantaurus-QY C11347-01 calibrated integrating sphere system (Hamamatsu Photonics K.K., Hamamatsu, Shizuoka, Japan).

### 3.3. Syntheses

Bispyrenyl malonate **1** was prepared by the synthetic routes depicted in Scheme 1. Disubstituted malonic acid dichloride (2-benzyl-2-methyl-malonyl dichloride) was prepared by the same method reported previously [49]. Subsequently, the reaction of the disubstituted malonic acid dichloride with 1-pyrenylmetanol in benzene gave a desired bispyrenyl malonate **1**. Bispyrenyl malonate **2** was prepared by similar method.

1-Pyrenylmethanol (4.6 g) was dissolved in dry benzene (200 mL). Dry pyridine (3.0 mL) was added to the solution and stirred for 1 h. In a dark room, the dry benzene solution (35 mL) of 2-benzyl-2-methylmalonyl dichloride (2.4 g) was added dropwise to the solution. The reaction solution was stirred for 24 h at room temperature and refluxed for 72 h. 0.5 M hydrochloric acid aqueous solution (120 mL) was added to the reaction solution. The solution was extracted with chloroform (100 × 3 mL). The organic layer was dried over anhydrous magnesium sulfate, filtrated, and evaporated under reduced pressure. The residue was thoroughly washed with hexane and methanol. The purification was performed by liquid chromatography (CHEMCOSORB 5-ODS-H) with methanol–chloroform (8.5:3), to obtain bis(1-pyrenemethyl)-2-benzyl-2-methylmalonate **1** (4.4 g, 66% isolated yield); pale yellow crystal; IR (KBr): 1737.7, 1272.9 cm^-1^. ^1^H-NMR (300 MHz NMR, CDCl_3_): 1.39 (s, 3H), 3.25 (s, 2H), 5.62 (d, 2H, *J* = 12.90 Hz), 5.67 (d, 2H, *J* = 12.90 Hz), 6.92–6.99 (m, 2H), 7.02–7.14 (m, 3H), 7.67 (d, 2H, *J* = 7.80 Hz), 7.80–7.96 (m, 12H), 8.01 (dd, 4H, *J* = 7.80 and 1.80 Hz). ^13^C-NMR (75 MHz NMR, CDCl_3_): 19.7, 41.1, 55.2, 65.4, 122.3, 124.2, 124.3, 124.4, 125.2, 125.2, 125.7, 126.8, 126.9, 127.1, 127.5, 127.9, 128.1, 128.8, 130.1, 130.3, 130.9, 131.3, 135.8, 171.6. HRMS (EI+): *m*/*z* calcd. for C_45_H_32_O_4_ 636.2301, found 636.2296.

1-Pyrenylmethanol (5.56 g) was dissolved in dry benzene (170 mL). Dry pyridine (5.5 mL) was added to the solution and stirred for 1 h. In a dark room, the dry benzene solution (30 mL) of 2-methyl-2-naphthalenylmethyl-malonyl dichloride (3.50 g) was added dropwise to the solution and stirred for 72 h at room temperature. The reaction solution was filtrated and evaporated under reduced pressure. 0.5 M hydrochloric acid aqueous solution (30 mL) was added to the residue. The solution was extracted with chloroform (100 × 3 mL) and washed with water (50 × 2 mL). The organic layer was dried over anhydrous magnesium sulfate, filtrated, and evaporated under reduced pressure. The residue was washed with hexane, ethyl acetate–hexane (1:1), and methanol to gain the crude yellow solid. The purification was performed by liquid chromatography (CHEMCOSORB 5-ODS-H) with ethyl acetate-hexane (1:20), to obtain bis(1-pyrenylmethyl)-2-methyl-2-naphthalenylmethyl-malonate **2** (0.17 g, 2% isolated yield); yellow crystal; IR(KBr): 1737.9, 1234.4 cm^−1^. ^1^H-NMR (400 MHz NMR, CDCl_3_): 1.45 (s, 3H), 3.43 (s, 2H), 5.67 (dd, 4H, *J* = 12.6 and 18.0 Hz), 7.04–7.70 (m, 7H), 7.77–8.05 (m, 18H). ^13^C-NMR (100 MHz NMR, CDCl_3_): 20.0, 41.4, 55.4, 65.6, 122.4, 124.3, 124.4, 124.5, 125.3, 125.3, 125.5, 125.8, 125.8, 127.1, 127.2, 127.4, 127.5, 127.6, 128.0, 128.3, 128.9, 129.0, 130.4, 131.0, 131.5, 132.3, 133.2, 133.4, 171.7. HRMS (FAB+): *m*/*z* calcd. for C_49_H_34_O_4_ 686.2457, found 686.2452.

### 3.4. X-ray Crystallographic Analysis

The suitable single crystal of bispyrenyl malonate **1** was obtained on slow solvent evaporation of the mixed solutions of methanol and chloroform. A measurement for bispyrenyl malonate **1** was made on a Rigaku R-AXIS RAPID diffractometer (Rigaku Corporation, Akishima, Tokyo, Japan) using graphite monochromated Cu Kα radiation. The structure of bispyrenyl malonate **1** was solved by direct methods [54] and expanded using Fourier techniques. A calculation was performed using the Crystal Structure [55] crystallographic software package except for the refinement, which was performed using SHELXL Version 2014/7 (http://shelx.uni-ac.gwdg.de/SHELX/) [56]. The non-hydrogen atoms were refined anisotropically. The H atoms were refined using the riding model. Crystallographic data are summarized in Table 1. Crystallographic data for the structures reported in this paper have been deposited with the Cambridge Crystallographic Data Centre as supplementary publication no. CCDC–1570537 for **1**. Copies of the data can be obtained free of charge on application to CCDC, 12 Union Road, Cambridge CB2 1EZ, UK [Fax: (internat.) +44-1223/336-033; E-mail: deposit@ccdc.cam.ac.uk].Suitable crystals for X-ray studies of derivative **2** and the corresponding complexes with the Cu^2+^ ions could not be obtained.

### 3.5. UV-Vis and Fluorescence Spectroscopy

UV-visible absorption spectra and fluorescence emission spectra were recorded at room temperature. A 1 × 1 cm quartz cuvette was used for the spectroscopic analysis. The stock solution (100 μM) of bispyrenyl malonate **1** in CH_3_CN/CHCl_3_ (9:1, *v*/*v*) was prepared for UV–visible and fluorescence spectroscopic analysis and diluted to a final concentration of 10 μM by mixing 10 μM stock solutions of inorganic perchlorates (LiClO_4_, NaClO_4_, NH_4_ClO_4_, Mg(ClO_4_)_2_, Cr(ClO_4_)_3_, Mn(ClO_4_)_2_, Fe(ClO_4_)_2_, Co(ClO_4_)_2_, Ni(ClO_4_)_2_, Cu(ClO_4_)_2_, Zn(ClO_4_)_2_, AgClO_4_, and Cd(ClO_4_)_2_). CH_3_CN/CHCl_3_ solutions of inorganic perchlorates were added to the solution of bispyrenyl malonate **1** that corresponded to 1 equiv. of metal ions. Although the maximal absorption peak of bispyrenyl malonate **1** is located at 342 nm, the excitation wavelengths at 344 nm was chosen for fluorescence spectroscopic analysis because the fluorescence intensity of the pyrene excimer emission at 467 nm became strongest. The emission spectra from ca. 350 to 770 nm were collected (every 1 nm). Excitation and emission slits width were 5 nm.

## 4. Conclusions

A novel bispyrenyl malonate compound **1** was successfully synthesized and its molecular structure confirmed by X-ray crystallographic analysis. Bispyrenyl malonate **1** crystallizes in the orthorhombic space group *P*2_1_2_1_2_1_ (no. 19). In the crystal structure of bispyrenyl malonate **1**, there are intramolecular C–H···π interactions between the hydrogen atoms on the pyrenyl C(32) and C(34) atoms and π-electrons of the other pyrenyl ring. The interaction distances between the hydrogens and pyrenyl moiety were 2.58 and 2.60 Å, respectively. In the acetonitrile/chloroform (9:1, *v*/*v*) solution, bispyrenyl malonate **1** exhibits an intramolecular excimer emission (λ_em_ = 467 nm) arising from the non-covalent C–H···π interactions between the pyrenyl moieties. Furthermore, the two pyrenyl moieties also participate in intermolecular π···π interactions with each of their neighboring molecules. The interplanar distance of the two pyrenyl moieties in a face-to-face manner was ca. 3.5 Å. The absolute fluorescence quantum yields of bispyrenyl malonate **1** in solution and in the solid state were Φ_PL_ = 0.13 and 0.65, respectively. The thus-obtained bispyrenyl malonate **1** acted as a highly selective fluoroionophore for the Cu^2+^ ion. Such enhancement of the fluorescence intensity of bispyrenyl malonate **1** was observed only in selective Cu^2+^ ion sensing.

## Figures and Tables

**Table 1:** X-ray crystallographic data for bispyrenyl malonate **1**.

Formula	C_45_H_32_O_4_
Formula weight	636.75
Crystal system	Orthorhombic
Space group	*P*2_1_2_1_2_1_ (no. 19)
*a*, Å	9.50566(17)
*b*, Å	12.5344(2)
*c*, Å	27.0375(5)
α, deg	90
β, deg	90
γ, deg	90
*V*, Å^3^	3221.46(10)
*Z*	4
*D*_calc_, g cm^-3^	1.313
μ(Cu Kα), cm^-1^	6.569
*T*, °C	−1.0
λ(Cu Ka), Å	1.54187
*R*1 ^a^	0.0620
*wR*2 ^b^	0.1780

^a^ *R*1 = ∑‖*F*_o_| − |*F*_c_‖/∑|*F*_o_|. ^b^ *wR*2 = [∑*w*(F_o_^2^ − F_c_^2^)^2^/∑*w*(F_o_^2^)^2^]^1/2^.

**Table 2:** Selected bond lengths (Å) and angles (deg) for bispyrenyl malonate **1**.

**Bond Lengths**			
O(1)–C(1)	1.212(5)	O(2)–C(1)	1.348(5)
O(2)–C(2)	1.453(6)	O(21)–C(21)	1.208(5)
O(22)–C(21)	1.331(5)	O(22)–C(22)	1.463(5)
C(1)–C(41)	1.512(6)	C(2)–C(3)	1.502(6)
C(22)–C(23)	1.500(6)	C(21)–C(41)	1.542(6)
C(41)–C(42)	1.573(7)	C(41)–C(43)	1.512(7)
C(43)–C(44)	1.520(7)		
**Bond Angles**			
C(1)–O(2)–C(2)	115.8(3)	C(2)–C(3)–C(16)	121.5(4)
O(1)–C(1)–O(2)	122.3(4)	O(21)–C(21)–C(41)	122.0(4)
O(2)–C(1)–C(41)	112.0(3)	O(22)–C(22)–C(23)	108.9(3)
C(2)–C(3)–C(4)	118.4(4)	C(22)–C(23)–C(36)	121.1(4)
O(21)–C(21)–O(22)	124.9(4)	C(1)–C(41)–C(42)	108.4(4)
O(22)–C(21)–C(41)	113.0(4)	C(21)–C(41)–C(42)	105.6(4)
C(22)–C(23)–C(24)	118.7(4)	C(21)–C(41)–C(43)	111.7(4)
C(1)–C(41)–C(21)	107.0(4)	C(41)–C(43)–C(44)	113.4(4)
C(1)–C(41)–C(43)	110.7(4)	C(43)–C(44)–C(49)	119.6(4)
C(21)–O(22)–C(22)	115.7(3)	C(42)–C(41)–C(43)	113.0(4)
O(1)–C(1)–C(41)	125.6(4)	C(43)–C(44)–C(45)	121.3(5)
O(2)–C(2)–C(3)	108.9(4)		

## Supplementary Materials

The following are available online, Figure S1: Fluorescence response of bispyrenyl malonate **1** to the Cu^2+^ ions in the presence of competitive cations. Figure S2: Fluorescence response of bispyrenyl malonate **2**. Figure S3: ^1^H- and ^13^C-NMR, HRMS, and FTIR spectra of bispyrenyl malonate **1**. Figure S4: ^1^H- and ^13^C-NMR, HRMS and FTIR spectra of bispyrenyl malonate **2**.
