# Supplementary material for: Bis(1-pyrenylmethyl)-2-benzyl-2-methyl-malonate as a Cu2+ Ion-Selective Fluoroionophore
Source: Molecules. 2017 Aug 25;22(9):1415. doi: 10.3390/molecules22091415 (PMC6151551; doi:10.3390/molecules22091415)
Supplement: Supplementary file 1 [file molecules-22-01415-s001.pdf]

Supplementary Material

Bis(1-pyrenylmethyl)-2-benzyl-2-methyl-malonate as a Cu^{2+} ion-selective Fluoroionophore

Takayo Moriuchi-Kawakami[1,*], Youji Hisada[1], Akihisa Higashikado[1], Tsubasa Inoue[1], Keiichi Fujimori[1], Toshiyuki Moriuchi[2]

[1]*Department of Applied Chemistry, Faculty of Engineering, Osaka Institute of Technology, 5-16-1 Omiya, Asahi, Osaka 535-8585, Japan*

[2]*Department of Applied Chemistry, Graduate School of Engineering, Osaka University, 2-1, Suita Yamada-oka, Osaka 565-0871, Japan*

1. Fluorescence Response of bispyrenyl malonate 1.

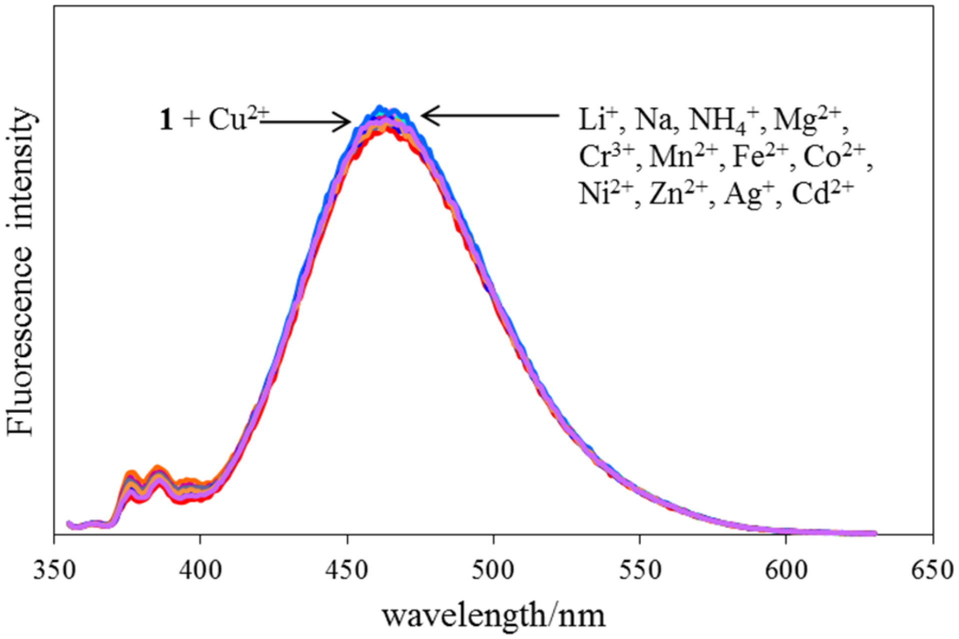

Fig. S1 Fluorescence spectra of bispyrenyl malonate **1** (10μM) mixed with 1 equiv. of the Cu^{2+} ions were changed in the presence of 1 equiv. of Li^{+}, Na^{+}, NH_4^{+}, Mg^{2+}, Cr^{3+}, Co^{2+}, Ni^{2+}, Cu^{2+}, Zn^{2+}, Ag^{+}, and Cd^{2+} in acetonitrile/chloroform (9:1, *v*/*v*) (excitation wavelength: λ_{ex} = 344 nm).

2. Fluorescence Response of bispyrenyl malonate 2.

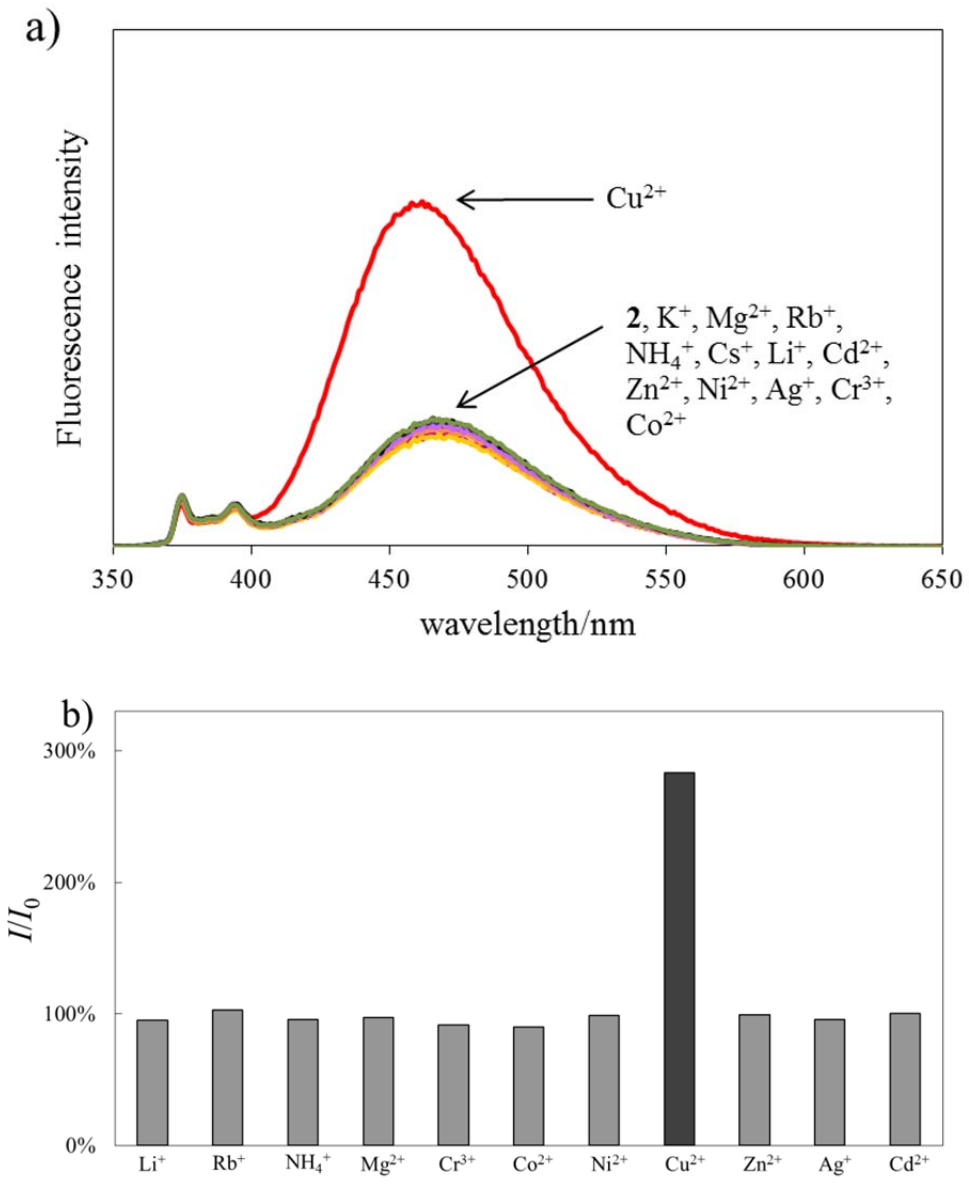

Fig. S2 (a) Fluorescence spectra and (b) ratio of the fluorescence intensities monitored at 464 nm of bispyrenyl malonate **2** (10μM) in the presence of 1 equiv. of Li^+, Rb^+, NH_4^+, Mg^{2+}, Cr^{3+}, Co^{2+}, Ni^{2+}, Cu^{2+}, Zn^{2+}, Ag^+, and Cd^{2+} in acetonitrile/chloroform (9:1, *v*/*v*) (excitation wavelength: λ_{ex} = 344 nm).

3. Spectral data of bispyrenyl malonate 1.

a)

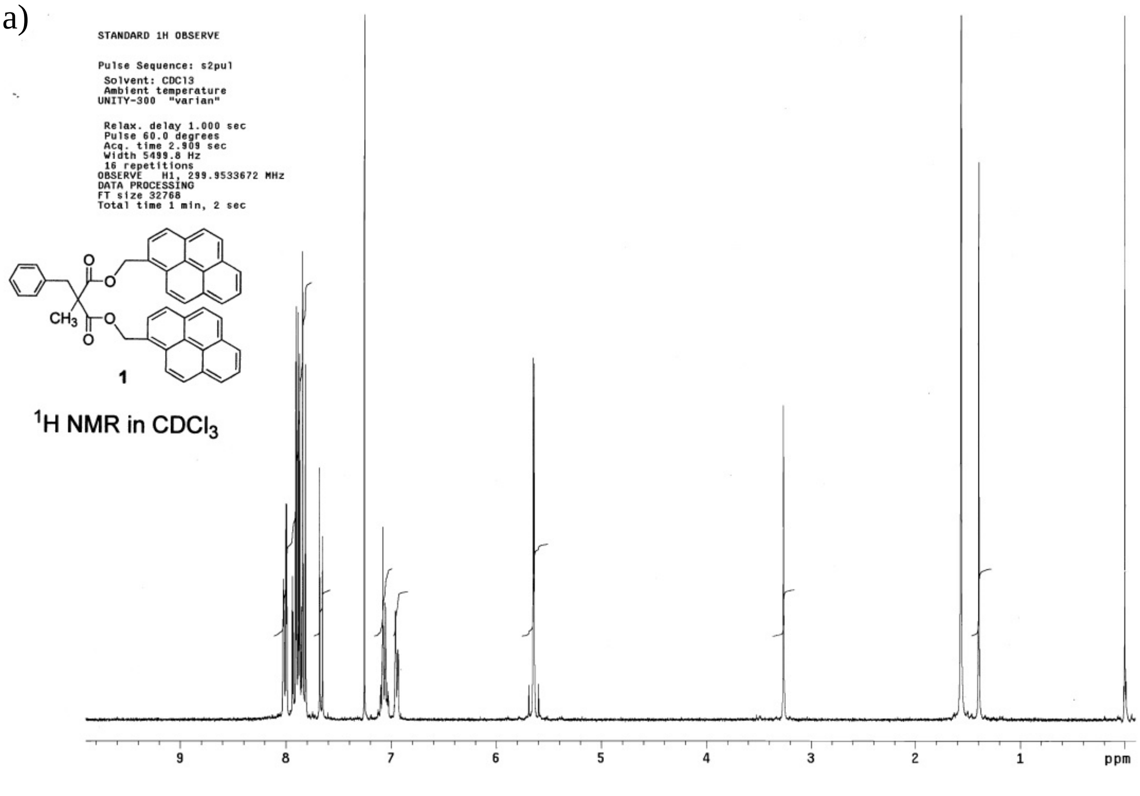

b)

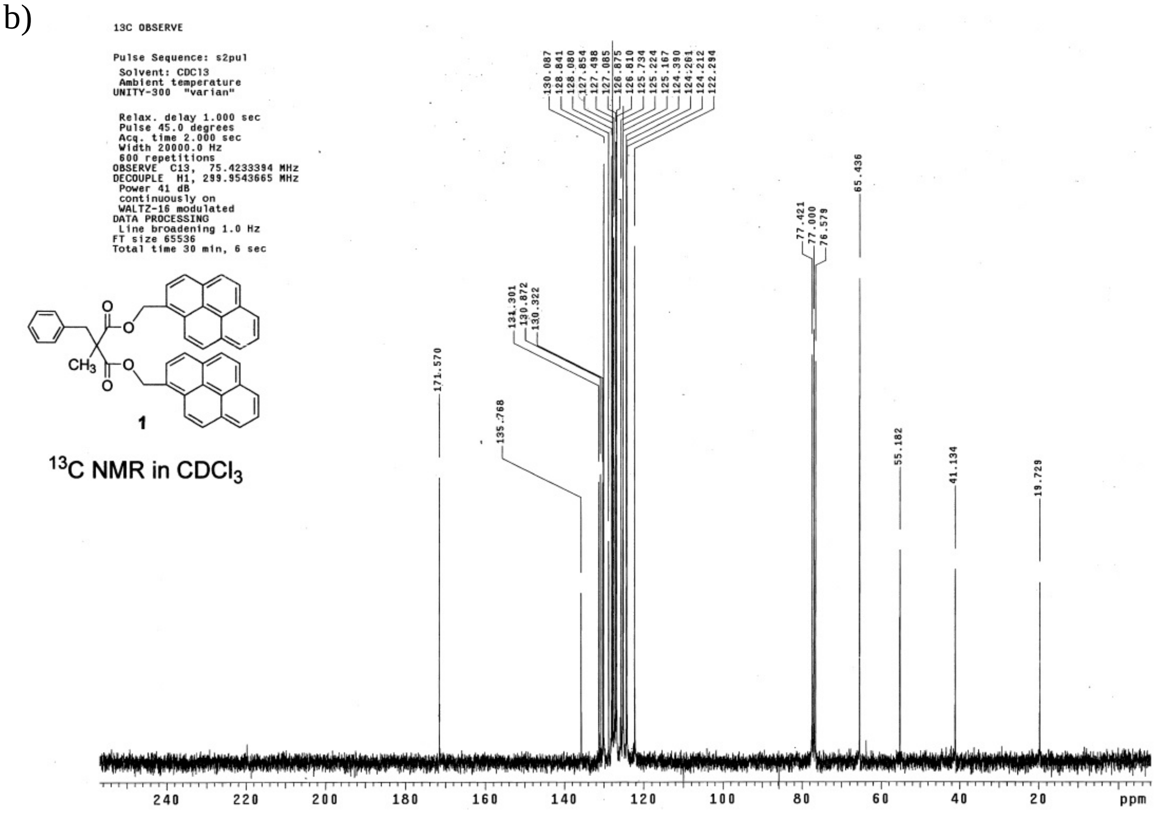

c)

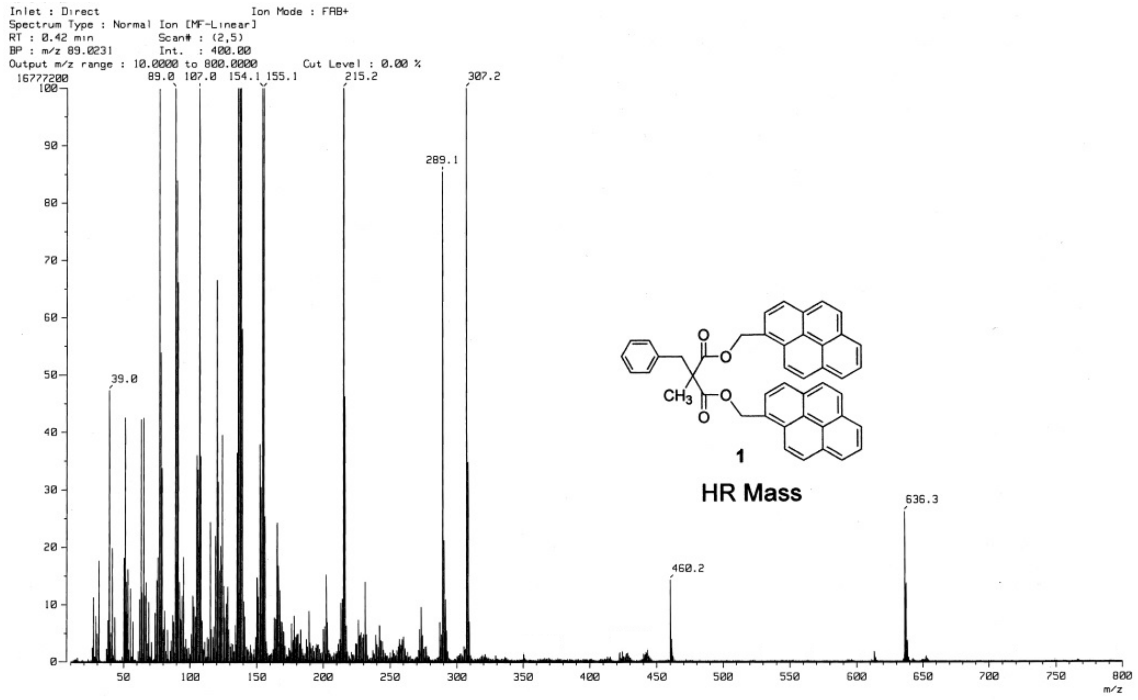

d)

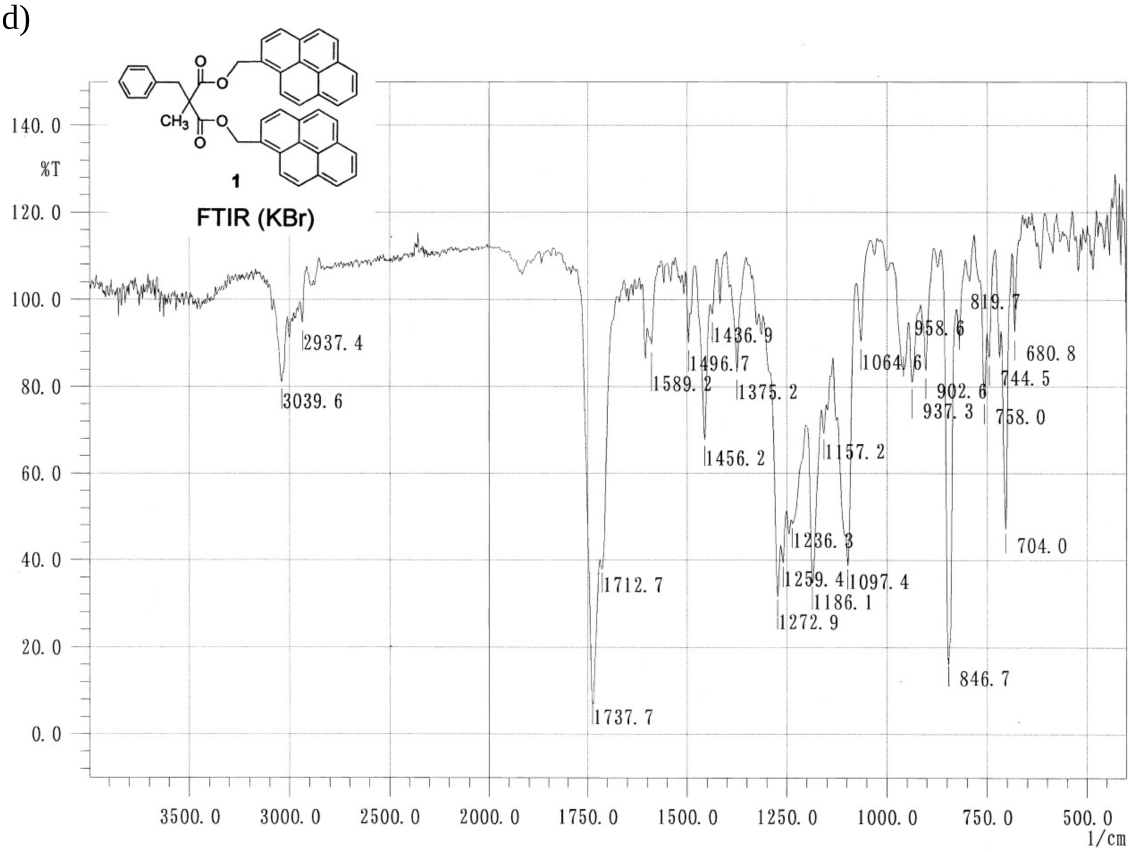

Fig. S3 (a) ^{1}H NMR and (b) ^{13}C NMR spectra, (c) HRMS and (d) FT IR of bispyrenyl malonate **1**.

***4.* Spectral data of bispyrenyl malonate 2.**

a)

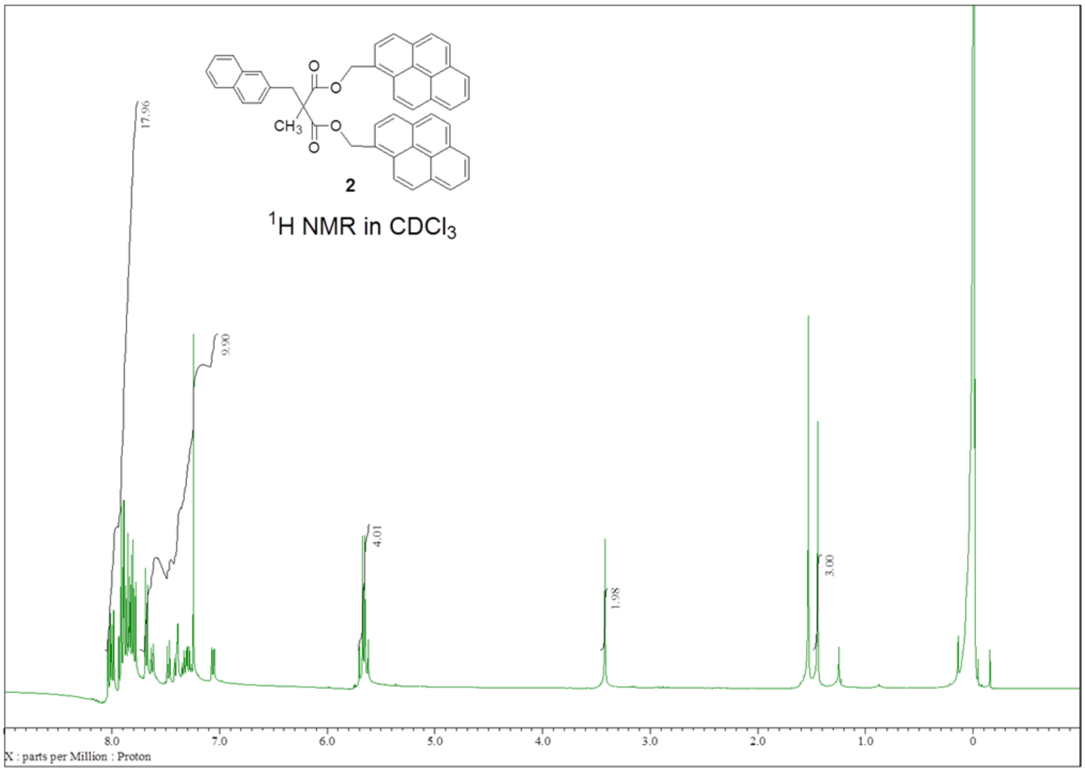

b)

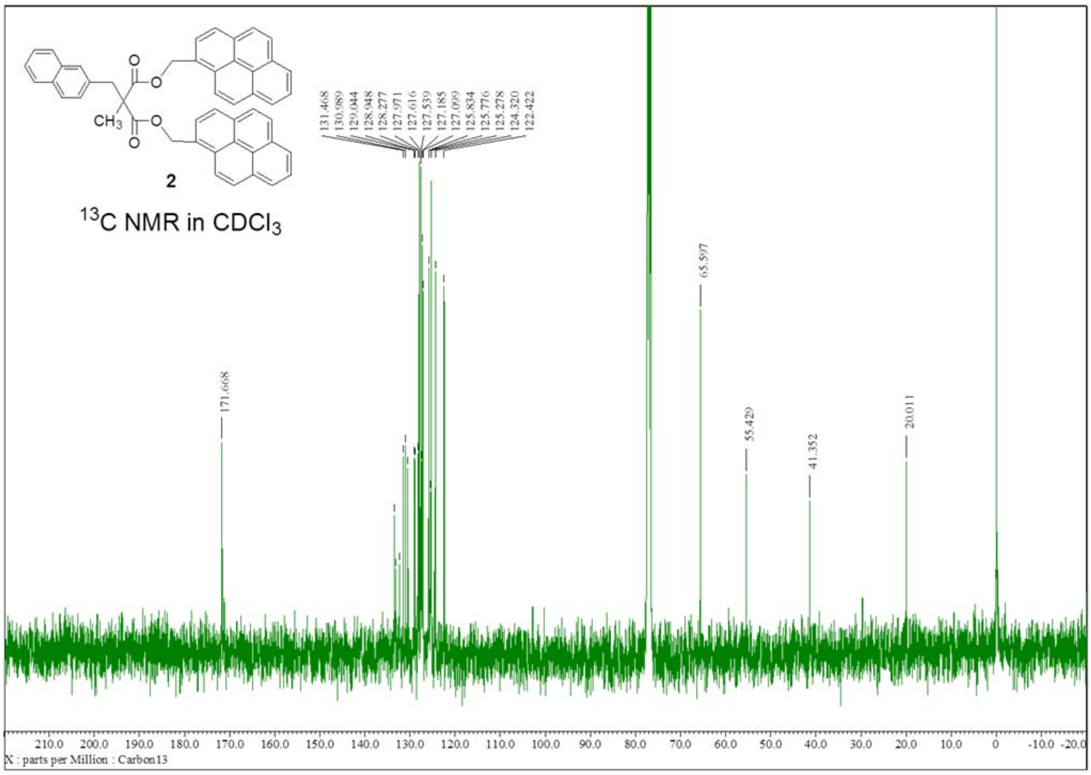

c)

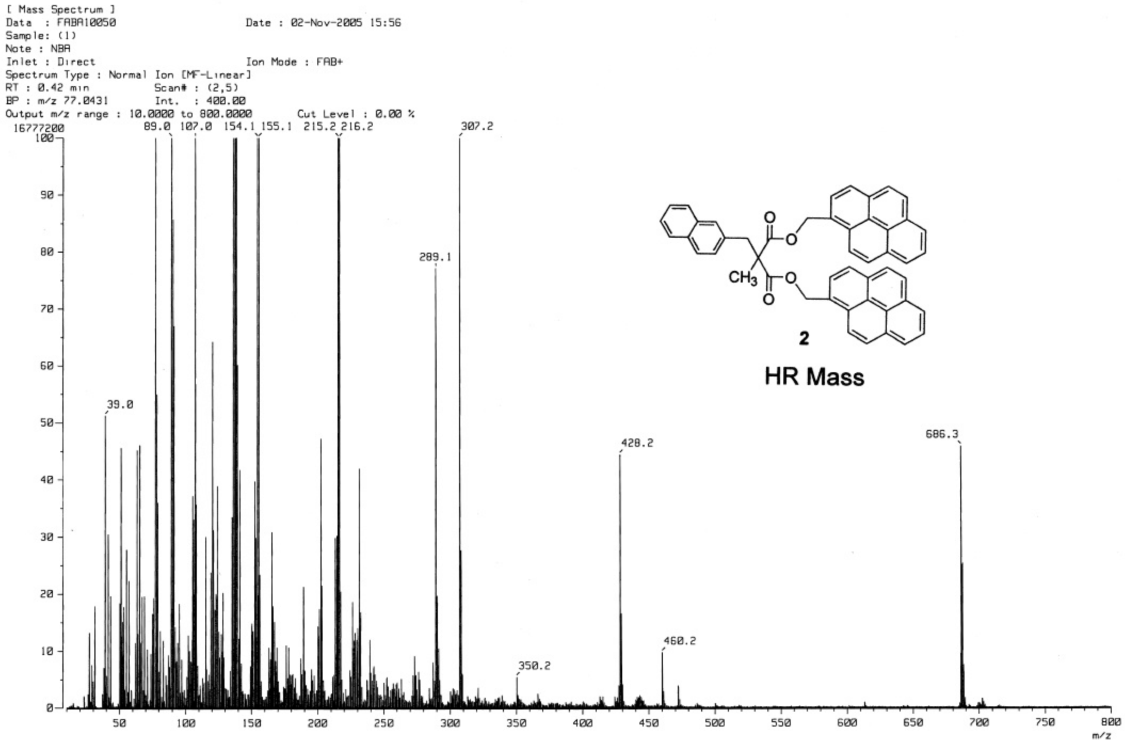

d)

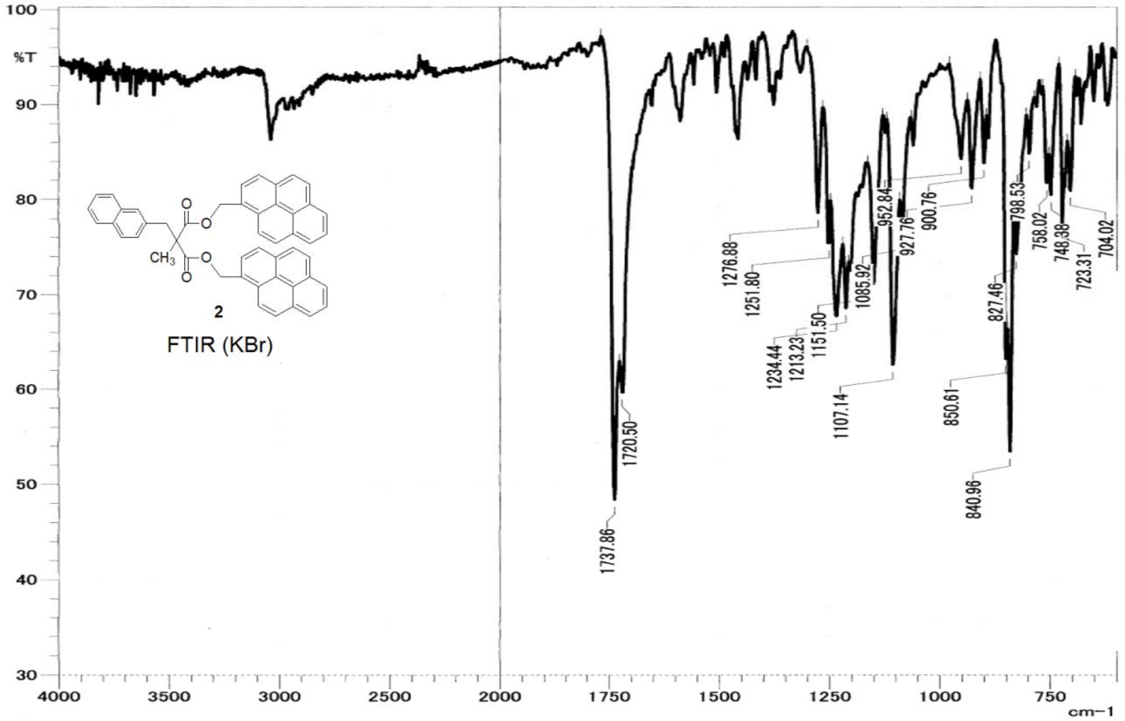

Fig. S4 (a) ^{1}H NMR and (b) ^{13}C NMR spectra, (c) HRMS and (d) FT IR of bispyrenyl malonate **2**.
